# Implementation of school tobacco policies: The advocacy coalition approach. Protocol of the ADHAirE study, a cluster randomized controlled trial

**DOI:** 10.18332/tpc/202392

**Published:** 2025-03-24

**Authors:** Pierre Laloux, Nora Mélard, Vincent Lorant

**Affiliations:** 1 Institute of Health and Society, Université Catholique de Louvain, Louvain, Belgium; 2 Foundation Against Cancer, Schaerbeek, Belgium

**Keywords:** smoking, school, policy, enforcement, adolescents, randomized

## Abstract

Secondary schools still struggle to enforce their tobacco policy and to keep their learning environment smoke-free. Yet, enforcement is the key to improve the effectiveness of those policies. This article describes the ADHAirE study which aims to reduce smoking at school through an improved enforcement of the school’s tobacco policy. The ADHAirE study will be carried out during 2 years in 20 Belgian secondary schools. Schools will be randomized either in the experimental or the control group. Schools in the experimental group will create an advocacy coalition involving students, staff and the principal. Those schools will also be linked to the others to share best practices about their school tobacco policy. Members of the advocacy coalition will share beliefs and values about the tobacco policy and about the school’s role in tobacco prevention. This randomized controlled trial will assess the effectiveness of the advocacy coalition to enforce the school tobacco policy. The ADHAirE study is based on the latest research and recommendations on school tobacco prevention. Following the social norm theory, this study goes further than many others before which only focused on health education, targeting the individual and not the social environment in which smoking occurs. Through the advocacy coalition, ADHAirE will initiate a community-level intervention that will ensure that all stakeholders are involved in decision-sharing about the rules, ensuring their acceptability, adoption and sustainability.

**CLINICAL TRIAL REGISTRATION:** The study is registered on the official website of ClinicalTrials.gov

**IDENTIFIER:** ID NCT06655038

## INTRODUCTION

Although the prevalence of adolescent smoking decreased since 2002 in the French-speaking part of Belgium, still 30% of those aged 14–18 years ever tried to smoke a cigarette, and 15% already smoke regularly, i.e. at least once a week^1^. This is close to the prevalence of daily smokers in the Belgian general population^2^. In addition, more than a third of these adolescents ever tried vaping, a behavior that increases the likelihood of initiating smoking cigarettes^1,3^.

School is considered an ideal setting to prevent smoking, as it plays an important role in influencing young people’s behavior and a large number of adolescents can be reached^4^. Yet, while research in that field has been going on since the 1980s (see details in the Supplementary file Material 1), no effective intervention to prevent adolescents from smoking in the long-term has emerged^5^. These interventions often target individual knowledge, skills, or behavior whereas smoking initiation mainly occurs through social interactions which take place in a wider social context (school, community, society etc.)^6^.

Accordingly, a strategy focused on the social environment and aiming to denormalize smoking seems to be appropriate to have a broader impact on adolescents’ smoking behavior. The introduction of school tobacco policies (STPs) which define whether or where adolescents and adults are allowed to smoke, but also penalties for those caught violating the rules^7^, were in principle promising to achieve a more successful prevention. They, actually, did not show strong evidence concerning their effectiveness in preventing youth tobacco use^8^.

To be effective, policies need to be comprehensive and well-implemented^9^. The latter often fails because of poor or inconsistent enforcement of the rules, which is an even more important feature of the policy’s effectiveness than its specific content^10^. A lack of commitment from the staff members because of different experiences, perceptions, and values may explain why STPs are not properly enforced, undermining their potential benefits. Staff members were found to be more inclined to enforce STPs if they felt supported by relevant actors in society such as parents, perceived health promotion as compatible with their professional identity, believed that STPs have positive outcomes on the staff–student relationship, were confident in the effectiveness of the policies, shared values of solidarity and social responsibility, and perceived smoking as a core problem^10,11^. Sharing beliefs toward school’s involvement not only in educational matters but also in health promotion could improve the enforcement of STPs.

Those results emerged from two waves of a European-wide observational study in schools, carried out in 2013 and 2016: the SILNE and SILNE-R studies. Those studies led to recommendations to improve the effectiveness of STPs. The continuing research on STPs highlighted the importance of: 1) Implementing comprehensive and clear rules prohibiting smoking for everyone, anytime and anywhere; 2) Communicating the purposes and the legitimacy of the policy; 3) Explicating staff roles and responsibilities; 4) Ensuring consistent enforcement with progressivity in disciplinary measures; and 5) Providing support for adolescents and staff members seeking to stop smoking or having difficulties to comply to the rules^12^. The need to focus on STPs to prevent smoking at school was part of recent interventional studies carried out in Denmark (X:IT, Focus, Smoke-Free Vocational Schools) ^13-15^, but they did not show significant decreases in smoking prevalence among students^16,17^. Those studies included STPs in a multicomponent approach beside educational courses, trainings towards staff, and other activities.

Based on these studies, we developed ADHAirE (A Decent and Healthy Air for Everyone), a school-based intervention to prevent smoking. To go further, ADHAirE will include the adolescents and the staff members in the decision-making process of the policy. By considering the point of view of the main stakeholders, we aim to improve the implementation of the STP by a better enforcement of the school staff, but also a better compliance to the rules by the students, as it is they who will have designed the STP. By embedding the STP in the schools’ routine practice, the final goal of ADHAirE is to guide schools to become smoke-free environments where adolescents are not exposed to smoking. In this article, smoking will refer to all products related to the tobacco industry. So, vaping, cannabis and others tobacco products are considered.

## METHODS

The study protocol complies with the SPIRIT (Standard Protocol Items: Recommendations for Interventional Trials) guidelines (see Supplementary file Material 2) and the CONSORT statement for cluster randomized trials.

### Program theory

**Figure 1:**
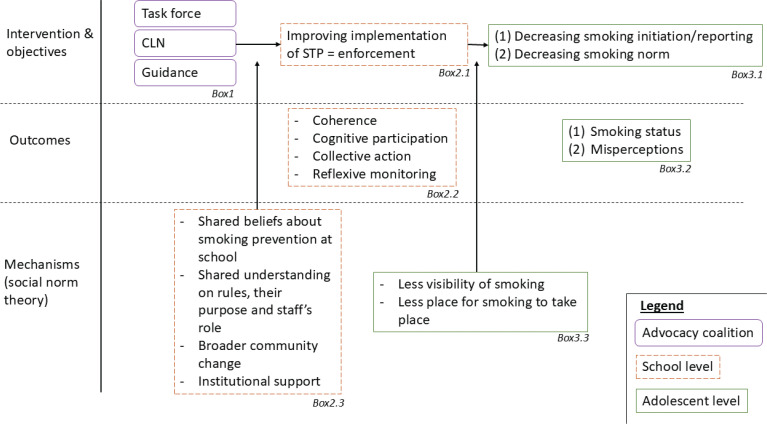
Program theory of the ADHAirE study

The intervention was built based on the Intervention Mapping Approach and its taxonomy of Behavior Change Methods^18^. As well as the Intervention Mapping Approach, we acknowledge that behavior is not only a function of individuals but also of the physical, social, and organizational environments in which they live. In this view, the ADHAirE study is based on the theory of social norms to explain the wider community behavior change^19^. Figure 1 shows the program theory.

Social norms are rules and standards that are understood by members of a group, and that guide and/or constrain social behavior without the force of law. They are driven by social expectations, i.e. expectations we have about other people’s behavior and beliefs^19^. In the case of smoking, misperceptions of what others do and approve of can lead adolescents to follow a ‘smoking norm’ that does not actually exist^19^.

To change a norm, a school needs to gather enough support advocating for not smoking at school. It needs an advocacy coalition of students, staff, and parents sharing the belief that schools should care to be a smoke-free environment. To achieve this, school stakeholders should rely on values of social responsibility. To be effective, the STP itself needs to be a social norm, shared, and enforced by a large coalition of school stakeholders. ADHAirE aims to shift norms within each school, positively promoting a safe and healthy tobacco-free environment for adolescents. So, smoking should be considered a matter of health and wellbeing rather than a matter of discipline, by involving all stakeholders in the development of that environment and providing schools with information and incentives to help shift attitudes and encourage support for a tobacco-free environment.

The advocacy coalition is a type of alliance that mobilizes diverse stakeholders who share common views about a specific policy. They agree on the core beliefs, values, and goals of this policy. They coordinate action and engage in joint strategies to influence policy-making^20^. If the advocacy coalition framework is usually focused on change processes over decades, shorter timeframes can also be considered^20^. Advocacy coalitions have already shown their impact on tobacco-control policy decision-making, mainly at European and national levels (United Kingdom, Japan, The Netherlands)^21^. The advocacy coalition framework usually enables to analyze the implementation process of a public policy^22^. Here we converted the framework into an intervention and adapted it to the organizational (school) level.

### Intervention

The intervention will take place during 2 school years, from September 2024 until June 2026. Participating schools will be given the objective of reaching a smoke-free school environment, i.e. smoking is forbidden for everyone, anywhere and anytime, and rules are enforced.

At least two project coordinators will be designated among the staff members (mainly teachers and educators) of each participating school. In Belgium, teachers are responsible for teaching, while educators are more dedicated to the social and disciplinary support of students. They serve as the primary point of contact for the research team to conduct the study in their school.

The intervention will consist of two main components:

1. Regular feedback to schools
2. The setting up of an advocacy coalition

*Feedback*

Regular monitoring of the school situation regarding adolescent smoking and its tobacco policy will serve to provide feedback to schools. This is one of the elements of the Nudging Theory of Thaler and Sunstein to facilitate decisions. This monitoring will be carried out through an audit of the school tobacco policy using a rating system, a survey of school staff attitudes and compliance regarding the policy, and a survey of adolescents’ smoking behaviors (see the Data collection section for more details). This will give the schools an overview of their situation, and of how this situation evolves. The feedback will be provided by the research team in a meeting with the principal after each collection of data. The principal can decide to invite more school actors to this meeting to broaden the number of recipients. This has been referred to as a formalized strategy of reflexive monitoring , defined as ‘the appraisal work that people do to assess and understand the ways that a new set of practices affect them and others around them’^23^. This regular monitoring is a minimal starting point to steer measures to take to achieve a smoking-free environment as gathering information on their student population can help schools to act upon it and take appropriate action^24^.

It might also serve to change factual beliefs, which is the first step suggested by Bicchieri^19^ to abandon a social norm. In our case, this might be the false belief that smoking is not common among adolescents anymore or the belief that the school tobacco policy is enforced at a sufficient level. Factual beliefs might be changed by presenting evidence that such beliefs are false. Moreover, regular monitoring might motivate school staff members if they realize that the school’s situation is positively evolving, leading to increased support for the school tobacco policy^11^.

*Advocacy coalition*

Besides the feedback, an advocacy coalition will be built. Three components (see Figure 1, Box 1) will be implemented to create this advocacy coalition:

1. A school taskforce
2. A collaborative learning and support network
3. Guidance

The school task force (TF)

Building a coalition requires a core group sharing and disseminating a common belief of why and how the school should improve its tobacco policy^25^. Shared understanding among school staff members and school authorities is a key factor for successful organizational implementation^26^. The task force aims to enable all actors to take ownership of the project and get actively involved^27^. Improved communication and social interaction, outcomes of this group, will be key to shift from a perceived norm to a collective norm (See Figure1, Box 2.3)^19^.

The TF will be set up in each school and includes various school actors such as students, teachers, educators, and the principal. These actors will meet 4 times a year to discuss, with the support of the research team (see Guidance below), the smoking situation in the school and adapt the rules to achieve a smoke-free school environment. Adult actors in the TF are the project coordinators and will be selected voluntarily. If a school council including students already exists, the TF will take place in this setting and smoking issues will be debated in addition to the already discussed topics. Otherwise, one or two students from each school year and each educational track can participate voluntarily in the TF.

The collaborative learning and support network (CLN)

One mechanism through which a social norm spreads is through social learning processes^28^. Those processes allow us to copy the most effective ways of doing and the most successful others. To help this process to happen, we will set up a CLN, also referred to as communities of practice, that will improve coordination between participating schools and extend the advocacy coalition beyond the school community. The collaborative learning network is a well-known approach to implement innovations and improve their quality. Given that the intervention occurs in a group setting, it makes sense to build new social networks to strengthens its effects.

The CLN will take place in the form of an online platform that allows the coordinators from each school to share the good practices about the implementation of STPs. It will also reinforce shared beliefs, create a common culture, social support and shared resources. The way of sharing information and the support to do it has to be discussed in collaboration with the schools. Moreover, a biannual meeting will bring the members of the CLN together to improve connections within the network.

Guidance

Guidance will consist of two parts. First, schools will be supported in the implementation of STPs and getting actors on board. The TF meetings will be led by members of the research team who will give evidence-based input and guide the process toward a smoke-free school. In concrete terms, the research team will help by deconstructing beliefs with the school actors, giving them advice on how to develop an effective STP, which components need to be included, how to graduate the sanctions in case of rule violation, how to communicate the rules and their rationale. Also, local health promotion organizations will be available to help the school to achieve the smoke-free school goal. This component will also include reflection on how to best fit the policy to the school’s context and around possible environmental changes. Fitting the policy resonates with the concept of re-invention, which is ‘the degree to which an innovation is changed or modified by a user in the process of its adoption and implementation’. This will help prevent schools from dropping out and will increase the sustainability of the project.

Second, schools will be supported to help adolescents and staff members who want to stop smoking. The School Health Promotion (*Promotion de la Santé à l’Ecole*, PSE) will help by referring these people to smoking cessation programs or a tobacco specialist. Although there is limited evidence of the effectiveness of smoking cessation interventions on young people and no evidence of their long-term effectiveness, adolescents are generally unaware of their level of nicotine dependence, so encouraging them early to see a tobacco cessation specialist might be a good option to have them reflect on their smoking habit. School staff members will also be referred to smoking cessation services if they are willing to stop smoking. For those who do not wish to stop smoking, the aim will be to guide them in a reflection regarding their tobacco consumption and their role towards adolescents. Such guidance will help them gain confidence in their legitimacy to enforce the policy and discuss smoking with adolescents in an open and honest manner, even if they themselves are smokers^11^.

### Design

**Figure 2:**
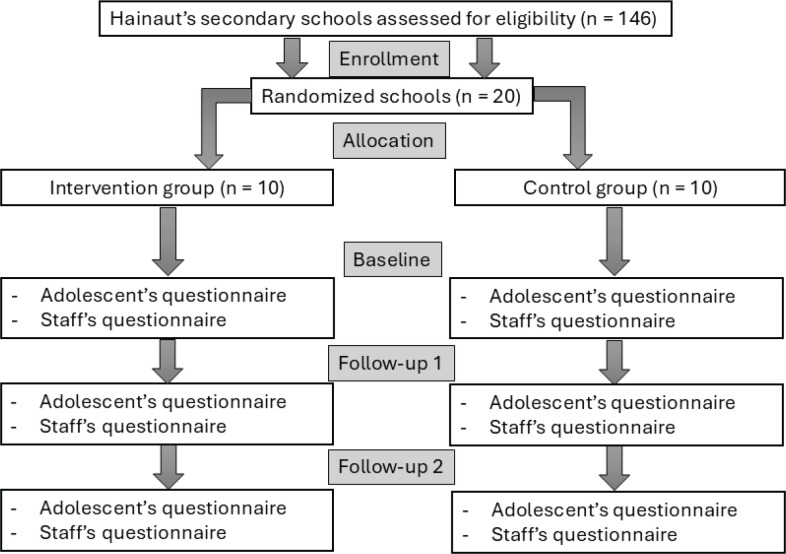
Flow chart of randomization and evaluation, ADHAirE study

The evaluation of the intervention is conducted as a cluster randomized controlled trial. Once voluntary schools had confirmed their willingness to participate, they were randomized to either the experimental arm or the control arm. Randomization follows a cross-stratification based on schools’ location, to avoid contamination effects, and their social-economic index, as the social-economic status is strongly associated with smoking. Figure 2 shows the flow chart displaying the research units per intervention arm.

*Control arm*

The SILNE and SILNE-R studies have shown that, without specific intervention, school tobacco policies remain unchanged over time^9^. To give all participating schools the opportunity to improve their smoking situation, even schools assigned to the control arm will receive minimal intervention: feedback based on the evaluation conducted. This point was also important to motivate schools to participate in a study where they had only one chance in two to get an intervention. To achieve the smoke-free school environment goal, these schools are then left on their own.

*Intervention arm*

Schools assigned to the intervention arm will benefit from the feedback and the advocacy coalition to achieve the smoke-free school environment goal.

### Study setting

The study takes place in Belgium, which has one of the highest inequality levels regarding smoking and lung cancer among OECD (Organization for Economic Cooperation and Development) countries, with people with a higher level of education having a longer life expectancy than those with a lower level of education^29^.

For this purpose, we target the Hainaut province which has one of the highest percentages of smokers (Hainaut: 22%, Belgium: 19%) and one of the highest rates of poverty in the country (Hainaut: 19%, Belgium: 13%)^2^. Also, focusing on one specific province facilitates contact with local stakeholders, dissemination of the results, and reduction in the implementation costs and logistical issues.

The Hainaut province is part of the French-speaking part of Belgium, where smoking on school premises has been prohibited since 2006. Smoking is prohibited in the rooms even when students are absent, and this extends to the open areas within the premises. However, the decree stipulating this is vague, it does not target specifically to whom the rules apply or when they apply. Also, it leaves schools a lot of room for implementation. No assessment was conducted since it was ratified. Moreover, there is a discrepancy with a law entered into force in 2010 about workers’ protection against tobacco smoke: this law allows employers to install a smoking room at the workplace.

### Recruitment

Schools were invited to participate in the study by letter in May 2024. The School Health Promotion (*Promotion de la Santé à l’Ecole*, PSE) and the education authority (*Fédération Wallonie-Bruxelles*) provided support in the dissemination of the invitation. After a few weeks, e-mails were sent as reminders to answer, then phone calls were made.

### Sample size

*School level*

We will recruit a total of 20 schools: 10 for the experimental group, and 10 for the control group, similar to previous European studies on similar topics^15^.

*Adolescent level*

Due to the clustering by school, the adolescent sample size must be inflated using a design effect so that school-specific smoking rates or school tobacco policy implementation can be considered. This design effect is equal to 1+(n-1)/ρ where n is the average school size (600 students in that province) and ρ is the intraclass correlation of smoking (0.05% in the SILNE study). As we will only evaluate adolescents from the 3rd and 4th academic years, we aim to survey 200 students per school. We expect a modest relative difference of 15% in smoking initiation between the experimental group and the control group, resulting in a total sample size of 2891 individuals.

### Outcomes

*School level*

When implementing an intervention, not only the effectiveness of the intervention has to be assessed, but also the extent to which it has been implemented. Overlooking this could lead to a Type III error, known as a conclusion that erroneously attributes observed findings to the intervention while this latter was not implemented as planned. Therefore, we will assess the fidelity of key elements of the intervention (the TF, the CLN, and the Guidance). Questionnaires used in previous school-based tobacco prevention interventions will be used^25^.

Two main outcomes will be considered, readiness for change and normalization of the intervention. Readiness for change is known as the ability and willingness of an organization to implement an innovation and is an indicator of successful implementation^30^. As this can influence the impact of the intervention and also be used as a tool to build on an organization’s readiness, we will measure it following the work done in the X:IT study^13^. Also, the normalization of the intervention will be a focal point as the implementation process ends when the intervention is embedded in an organization’s routine, one of the objectives of the study^25^. The Normalisation Process Theory will help us to understand the mechanisms underlying the shift from the STP to a social norm, shared by various actors across the board (See Figure 1, Box 2.3).

*Adolescent level*

Primary outcomes

The social norms about smoking will be assessed. Key indicators will be the change in the descriptive norm (or the perception of how many students smoke at school) and in the injunctive norm (or the perception of others approval of smoking).

We will mainly focus on misperceptions held by adolescents about peers’ smoking/vaping status and their approval towards smoking as they may be a leading cause of the maintenance of a smoking norm (See Figure1, Box 3.2).

Secondary outcomes

Smoking status will be key indicators of adolescents’ behavior. They will be based on the amount and frequency of smoking (See Figure1, Box 3.2).

Other data

Data will also be collected to gather information on the socio-economic determinants of the adolescents such as gender, age, or their perceived social-economic status but also on their perception of the school climate and their identification with the school.

### Statistical analysis

The effect of the advocacy coalition on primary and secondary outcomes will be analyzed by multilevel regression models. Those account for the nested hierarchical structure of the data (per school). Subgroup analyses will be carried out to evaluate the differential effect of the intervention based on sociodemographic factors at the individual and at the school level. Sensitivity analysis will also evaluate the association between the degree of implementation and the effect of the intervention.

### Data collection

The evaluation will take place three times: at baseline (September 2024), at follow-up after 1 year (September 2025), and at follow-up after two years (September 2026). We will gather information in three different ways: an audit, a survey for the adolescents, and one for staff members.

*Audit*

The audit will evaluate the school tobacco policy itself using a questionnaire completed by the principal. It will be addressed to the project coordinators of each school and covers the comprehensiveness, enforcement, and communication of the policy but also the provision of services, in the form of support to smoking cessation and smoking prevention activities.

*Adolescents’ survey*

The adolescents’ survey will be carried out following a repeated cross-sectional design. So, at each data collection, adolescents from the 3rd and 4th academic years will complete the survey. As adolescents will, during the 2 years of the intervention, move on through their academic course, survey participants will differ from year to year. This design is commonly used when an intervention aims to affect a community-level indicator of health.

*Staff survey*

As teachers have been referred to as the main agents and change makers in school health programs, it is critical to get an insight into their perception of the school’s duty to integrate health promotion into its policy. Also, social processes such as change agents, their relationships, and the context are important when implementing a program and will be assessed.

## DISCUSSION

This article describes the protocol of a cluster randomized controlled trial that aims to assess the effectiveness of an innovative way to implement STP in order to reduce adolescent smoking and vaping. The setting-up of an advocacy coalition in a school has a lot of potential to help effectively implementing STP and further decrease adolescent smoking.

First, we followed the recommendations that Galanti et al.^8^ formulated in their review of school tobacco policies. They highlighted promising components (comprehensiveness, communication, enforcement, and consequences) on which ADHAirE is based to improve schools’ smoking policy. They also underscored the need for longitudinal and experimental studies on the topic to strengthen the evidence about STPs effectiveness^8^.

Second, it is important to see the ADHAirE study as a long-term effort, and as an ongoing social process rather than as a one-time intervention. The aim of this project is not to overwhelm adolescents with multiple activities to encourage them not to smoke, it is rather a deep remodeling of the school’s position regarding smoking prevention and health promotion in general. This contrasts with most studies in the field that mainly focus on adolescents’ individual skills improvement such as perceived control or awareness of social influence. Few of these showed significant results in the long-term^5^. Also, to redirect adolescents’ social norms and behaviors, ADHAirE not only targets the social environment but also the physical environment as a source of social exposure. Both provide information for the development of norms but the latter serves as a cue for the former and is only rarely addressed.

Third, the advocacy coalition will integrate staff members and adolescents into the decision-making process. Findings from the European Network of Health Promoting Schools, a program that sought to integrate policy and practice of health in the educational sector, recognized teachers’ involvement as crucial for the outcomes of the program^31^. They are the main contributors in the implementation process of school-based programs, whether in the adoption phase or the embeddedness of the innovation in the school’s routine, but even in its sustainability. Andersen et al.^17^ discussed that the lack of effect of the X:IT study at its second follow-up might be caused by the lack of sustainability of the project. The research team had close contact with the schools during the first year and obtained positive results but had little to no contact with schools in the second year. We believe that the creation of a school task force and a collaborative learning and support network might help to ensure the sustainability of the project throughout its duration and even after the end. Teachers are also important role models for adolescents, therefore contributing to the legitimation or not of smoking^32^. Therefore, securing their buy-in and overcoming their resistances in a smoking prevention intervention is key to shifting adolescents’ perceived acceptability of smoking.

Fourth, ADHAirE draws on the social norms theory which is particularly adapted to situations of innovation. Deviating from a common practice can be costly in terms of social sanctions. So, people have to share reasons to behave differently and be sure they are not acting alone towards the new behavior. Group coordination can help to shift normative expectations and reduce the consequences of deviating from the ‘old’ norm^19^. The theory applies to both the intervention and the target group. First, the advocacy coalition is expected to carry out the project in such a way that STPs will become the new norm to prevent smoking at school. Second, reducing social exposure to smokers will reduce the visibility of the behavior and so shift empirical expectations, and thus the norm of smoking among adolescents^19^.

Lastly, the ADHAirE study is developed to ensure that all socio-economic groups feel included, to avoid differential effects. It targets change in the context and circumstances within which smoking occurs, and not the behavior in itself ^33^. In addition, ADHAirE is a community health intervention based on social responsibility associated with smoking and is not an intervention that seeks to change individual behaviors. Such strategy tends to contribute to narrowing social inequalities in smoking, as opposed to preventive interventions that rely more on individuals’ behavior change and are more likely to widen them^33^. Also, the intervention is implemented in a province with a high smoking prevalence and a low-income level compared to the country average. So, the intervention also targets a geographical area with the highest needs. Finally, from a methodological point of view, the sampling of the schools is stratified by the level of the school socio-economic status.

## CONCLUSIONS

The ADHAirE project is designed to address the topic of adolescent smoking as a community health issue. It is not limited to enforcing a smoking ban at school, as this may just lead to the displacement of the behavior outside school^9,34-36^. Instead, the project will guide schools to view their tobacco policy as a prevention tool and embed it so that it becomes a social norm shared and enforced by all, or in other words, as routine practice. This stratified randomized control trial will contribute to the understanding of school tobacco policies and provide further evidence of their effect on adolescent smoking.

## Supplementary Material



## Data Availability

Data sharing is not applicable to this article as no new data was created.
