# Supplementary material for: Implementation of school tobacco policies: The advocacy coalition approach. Protocol of the ADHAirE study, a cluster randomized controlled trial
Source: Tob Prev Cessat. 2025 Mar 24;11:10.18332/tpc/202392. doi: 10.18332/tpc/202392 (PMC11931910; doi:10.18332/tpc/202392)
Supplement: Supplementary file 1 [file TPC-11-20-s1.pdf]

Supplementary material 1 – other references used

Introduction

Aveyard, P., et al. (2004). "A methodological and substantive review of the evidence that schools cause pupils to smoke." Social science & medicine **58**(11): 2253-2265.

Bast, L. S., et al. (2019). "Study protocol of the X: IT II-a school-based smoking preventive intervention." BMC Public Health **19**(1): 1-11.

Hawe, P., et al. (2009). "Theorising interventions as events in systems." American journal of community psychology **43**: 267-276.

Peterson, A. V., et al. (2000). "Hutchinson Smoking Prevention Project: long-term randomized trial in school-based tobacco use prevention—results on smoking." JNCI: Journal of the National Cancer Institute **92**(24): 1979-1991.

Thomas, R. E., et al. (2015). "Effectiveness of school-based smoking prevention curricula: systematic review and meta-analysis." BMJ Open **5**(3): e006976.

Wiehe, S. E., et al. (2005). "A systematic review of school-based smoking prevention trials with long-term follow-up." Journal of Adolescent Health **36**(3): 162-169.

Social norm theory

Cialdini, R. B., et al. (1991). A focus theory of normative conduct: A theoretical refinement and reevaluation of the role of norms in human behavior. Advances in experimental social psychology, Elsevier. **24:** 201-234.

Kok, G., et al. (2016). "A taxonomy of behaviour change methods: an intervention mapping approach." Health Psychology Review **10**(3): 297-312.

Mead, E. L., et al. (2014). "Understanding the sources of normative influence on behavior: The example of tobacco." Social science & medicine **115**: 139-143.

Sargent, R. H. and L. S. Newman (2021). "Pluralistic ignorance research in psychology: A scoping review of topic and method variation and directions for future research." Review of General Psychology **25**(2): 163-184.

Advocacy coalition

Arnott, D., et al. (2007). "Comprehensive smoke-free legislation in England: how advocacy won the day." Tobacco control **16**(6): 423-428.

Fleury, M.-J., et al. (2014). "The role of advocacy coalitions in a project implementation process: The example of the planning phase of the At Home/Chez Soi project dealing with homelessness in Montreal." Evaluation and Program Planning **45**: 42-49.

Sabatier, P. A. (1988). "An advocacy coalition framework of policy change and the role of policy-oriented learning therein." Policy sciences **21**(2-3): 129-168.

Sato, H. (1999). "The advocacy coalition framework and the policy process analysis: The case of smoking control in Japan." Policy studies journal **27**(1): 28-44.

Weible, C. M. and K. Ingold (2018). "Why advocacy coalitions matter and practical insights about them." Policy & politics **46**(2): 325-343.

Willemsen, M. C. and J. V. Been (2022). "Accelerating tobacco control at the national level with the Smoke-free Generation movement in the Netherlands." npj Primary Care Respiratory Medicine **32**(1): 58.

Intervention

Berkowitz, A. D. (2005). "An overview of the social norms approach." Changing the culture of college drinking: A socially situated health communication campaign **1**: 193-214.

Bunger, A. C., et al. (2016). "Can learning collaboratives support implementation by rewiring professional networks?" Administration and policy in mental health and mental health services research **43**: 79-92.

Fanshawe, T. R., et al. (2017). "Tobacco cessation interventions for young people." Cochrane database of systematic reviews(11).

Gesell, S. B., et al. (2013). "Social network diagnostics: a tool for monitoring group interventions." Implementation Science **8**(1): 1-12.

Hjort, A. V. (2023). "Towards' smoke-free school hours' as part of routine practice: Examining implementation processes and outcomes related to the Smoke-Free Vocational Schools intervention."

Lapinski, M. K. and R. N. Rimal (2005). "An explication of social norms." Communication theory **15**(2): 127-147.

Rogers EM. Diffusion of Innovations, 5th Edition: Free Press; 2003.

Sugden, R. (2009). On nudging: A review of nudge: Improving decisions about health, wealth and happiness by Richard H. Thaler and Cass R. Sunstein, Taylor & Francis.

Vallata, A., et al. (2021). "Predictors of cigarette smoking cessation in adolescents: a systematic review." Journal of Adolescent Health **68**(4): 649-657.

Wenger, E. (1999). Communities of practice: Learning, meaning, and identity, Cambridge university press.

Design

Cheadle, A., et al. (1995). "Evaluating the usefulness for school principals of feedback reports from a school-based adolescent health survey." Evaluation review **19**(6): 675-686.

Eldridge, S. and S. Kerry (2012). A practical guide to cluster randomised trials in health services research, John Wiley & Sons.

James, K., et al. (2024). "Brief Educational Workshops in Secondary Schools Trial (BESST trial), a school-based cluster randomised controlled trial of the DISCOVER workshop for 16–18-year-olds: recruitment and baseline characteristics." Trials **25**(1): 302.

Keogh-Brown, M., et al. (2007). "Contamination in trials of educational interventions."

Lorant, V., et al. (2017). "Social network and inequalities in smoking amongst school-aged adolescents in six European countries." International Journal of Public Health **62**(1): 53-62.

Study setting

Décret relatif à la prévention du tabagisme et l'interdiction de fumer à l'école, (2006).

Loi du 22 décembre 2009 instaurant une réglementation générale relative à l'interdiction de fumer dans les lieux fermés accessibles au public et à la protection des travailleurs contre la fumée du tabac, (2009).

Sample size

Källmén, H., et al. (2020). "Effects of a school tobacco policy on student smoking and snus use." Health Behavior and Policy Review **7**(4): 358-365.

Rozema, A., et al. (2019). "Results of outdoor smoking bans at secondary schools on adolescents smoking behaviour: a quasi-experimental study." European Journal of Public Health **29**(4): 765-771.

Statbel. La Belgique en chiffres [Available from: https://statbel.fgov.be/fr].

Outcomes

Bast, L. S., et al. (2021). "School coordinators' perceptions of organizational readiness is associated with implementation fidelity in a smoking prevention program: Findings from the X: IT II study." Prevention Science **22**: 312-323.

Ferm, L., et al. (2018). "Operationalizing a model to quantify implementation of a multi-component intervention in a stepped-wedge trial." Implementation Science **13**(1): 1-14.

Kremers, S., et al. (2004). "Development and longitudinal test of an instrument to measure behavioral stages of smoking initiation." Substance use & misuse **39**(2): 225-252.

May, C. and T. Finch (2009). "Implementing, embedding, and integrating practices: an outline of normalization process theory." Sociology **43**(3): 535-554.

Murray, E., et al. (2010). "Normalisation process theory: a framework for developing, evaluating and implementing complex interventions." BMC medicine **8**: 1-11.

Data collection

Eldridge, S. and S. Kerry (2012). A practical guide to cluster randomised trials in health services research, John Wiley & Sons.

Jariego, I. M., et al. (2024). "Using personal network analysis to understand the interaction between programmes' facilitators and teachers in psychoeducational interventions." Evaluation and Program Planning: 102410.

Tjomsland, H. E., et al. (2009). "The Norwegian network of health promoting schools: A three-year follow-up study of teacher motivation, participation and perceived outcomes." Scandinavian Journal of Educational Research **53**(1): 89-102.

Discussion

Friend, S., et al. (2014). "The researchers have left the building: what contributes to sustaining school-based interventions following the conclusion of formal research support?" Journal of School Health **84**(5): 326-333.

Hjort AV. Towards 'smoke-free school hours' as part of routine practice: Examining implementation processes and outcomes related to the Smoke-Free Vocational Schools intervention: University of Southern Denmark; 2023.

Kjeld, S. G., et al. (2023). "From a teacher and school leader perspective: What happened with smoking rules and practices during a three-year smoking preventive intervention?–findings from the X: IT II study." Evaluation and Program Planning: 102236.

Lagerweij, N. A., et al. (2019). "The visibility of smoking in Europe and its relationship with youth's positive beliefs about smoking." International Journal of Public Health **64**: 1335-1344.

Mead, E. L., et al. (2014). "Understanding the sources of normative influence on behavior: The example of tobacco." Social science & medicine **115**: 139-143.

Nolan, J. M. (2017). "Social norms and their enforcement." The Oxford handbook of social influence: 147-164.

Rod, M. H., et al. (2014). "The spirit of the intervention: reflections on social effectiveness in public health intervention research." Critical Public Health **24**(3): 296-307.

Wanless, S. B. and C. E. Domitrovich (2015). "Readiness to implement school-based social-emotional learning interventions: Using research on factors related to implementation to maximize quality." Prevention Science **16**(8): 1037-1043.

Yazidjoglou, A., et al. (2024). "Electronic cigarette social norms among adolescents in New South Wales, Australia." Health Promotion International **39**(2): daae018.

Supplementary material 2 – SPIRIT reporting guidelines

Administrative information

Title

1. Descriptive title identifying the study design, population, interventions, and, if applicable, trial acronym

Title: Implementation of school tobacco policies: the advocacy coalition approach. Protocol of the ADHAirE study, a cluster randomised controlled trial

- Study design: cluster randomised controlled trial
- Population: schools
- Intervention: advocacy coalition

Trial acronym: ADHAirE

Trial registration

2a. Trial identifier and registry name. If not yet registered, name of intended registry

In abstract: NCT06655038 (ClinicalTrials.gov)

2b. All items from the World Health Organization Trial Registration Data Set

Data category	Information
Primary registry and trial identifying number	ClminicalTrials.gov NCT06655038
Date of registration in primary registry	23 October, 2024
Secondary identifying numbers	/
Source(s) of monetary or material support	Foundation against Cancer (Belgium)
Primary sponsor	Foundation against Cancer
Secondary sponsor(s)	/
Contact for public queries	PL
Contact for scientific queries	PL
Public title	Implementation of school tobacco policies: ADHAirE protocol
Scientific title	Implementation of school tobacco policies: the advocacy coalition approach. Protocol of the ADHAirE study, a cluster randomised controlled trial
Country of recruitment	Belgium
Health condition(s) or problem(s) studied	Smoking; Tobacco policies enforcement
Intervention	Intervention group:<br>- Feedback based on surveys<br>- Advocacy coalition<br>Control group:<br>- Feedback based on surveys
Key inclusion and exclusion criteria	School level:

	- Inclusion criteria: secondary school, located in Hainaut province<br>Students level:<br>- Inclusion criteria: 3rd-4th grade, in a recruited school
Study type	- Interventional<br>- Allocation: randomized, by cluster (school)<br>- Primary purpose: prevention
Date of first enrolment	1st May 2024
Target sample size	- 20 schools<br>- 2900 students
Recruitment status	Recruiting
Primary outcome	Smoking norm
Secondary outcome	Smoking prevalence

Protocol version

3. Date and version identifier

First submitted on ClinicalTrials.gov: 22-10-2024

First posted on ClinicalTrials.gov: 23-10-2024

Funding

4. Sources and types of financial, material, and other support

Foundation against Cancer (Belgium, Project n° CPR-2022/1882)

Roles and responsibilities

5a. Names, affiliations, and roles of protocol contributors

- FARES (Fonds des affections respiratoires asbl)
- Observatoire de la santé du Hainaut
- SEPT (Service d'Etude et de Prévention du Tabagisme)

All these organizations are specialized in health promotion and more specifically in smoking prevention. They support the research team in providing information on practical issues.

5b. Name and contact information for the trial sponsor

Els Decoster: EDecoster@stichtingtegenkanker.be

5c. Role of study sponsor and funders, if any, in study design; collection, management, analysis, and interpretation of data; writing of the report; and the decision to submit the report for publication, including whether they will have ultimate authority over any of these activities

The sponsor:

o provides financial support for the research project.

o ensures the scientific and financial monitoring of the project on the basis of reports submitted by the Researchers and the University.

o supervises the correct use of the grant by the Researchers and the University.

5d. Composition, roles, and responsibilities of the coordinating centre, steering committee, endpoint adjudication committee, data management team, and other individuals or groups overseeing the trial, if applicable (see Item 21a for data monitoring committee)

Vincent Lorant	UCLouvain	Principal investigator
Pierre Laloux	UCLouvain	Research assistant
Audrey Delaire	UCLouvain	Technical support
William D'hoore	UCLouvain	Member of the steering committee
Andrea Rozema	Tilburg University	Member of the steering committee
Maïte Verloigne	UGent	Member of the steering committee
François Alla	Université de Bordeaux	Member of the steering committee
Nora Mélard	Fondation contre le Cancer	Member of the steering committee
Sophie Pierard	Observatoire de la santé du Hainaut	Member of the steering committee
Karine De Jonghe	Observatoire de la santé du Hainaut	Member of the steering committee
Anne Naudin	Services de promotion de la santé à l'école (PSE)	Member of the steering committee
Gregory Jac	Fédération Wallonie-Bruxelles	Member of the steering committee
Romina Loria	FARES	Member of the steering committee
Cédric Migard	FARES	Member of the steering committee
Marie Housieaux	FARES	Member of the steering committee
Céline Corman	SEPT	Member of the steering committee

Introduction

Background and rationale

6a. Description of research question and justification for undertaking the trial, including summary of relevant studies (published and unpublished) examining benefits and harms for each intervention

...The need to focus on STPs to prevent smoking at school was part of recent interventional studies carried out in Denmark (X:IT, Focus, Smoke-Free Vocational Schools) [13-15], but they did not show significant decreases in smoking prevalence among students [16,17]. Those studies included STPs in a multicomponent approach beside educational courses, trainings towards staff, and other activities...To go further, ADHAirE will include the adolescents and the staff members in the decision-making process of the policy. By considering the point of view of the main stakeholders, we aim to improve the implementation of the STP by a better enforcement of the school staff but also a better compliance to the rules by the students, as it is they who will have designed the STP.

6b. Explanation for choice of comparators

The SILNE and SILNE-R studies have shown that, without specific intervention, school tobacco policies remain unchanged over time[9]. To give all participating schools the opportunity to improve their smoking situation, even schools assigned to the control arm will receive minimal intervention: feedback based on the evaluation conducted. This point was also important to motivate schools to participate in a study where they had only one chance in two to get an intervention. To achieve the smoke-free school environment goal, these schools are then left on their own.

Objectives

7. Specific objectives or hypotheses

ADHAirE's final goal is to guide schools to become smoke-free environments where adolescents are not exposed to smoking.

Trial design

8. Description of trial design including type of trial (eg, parallel group, crossover, factorial, single group), allocation ratio, and framework (eg, superiority, equivalence, noninferiority, exploratory)

The evaluation of the intervention is conducted as a cluster randomised controlled trial. Once voluntary schools had confirmed their willingness to participate, they were randomised to either the experimental arm or the control arm. Randomisation follows a cross-stratification based on schools' location, to avoid contamination effects, and their social-economic index, as the social-economic status is strongly associated with smoking. *Figure 2* shows the flow chart displaying the research units per intervention arm.

Methods: Participants, interventions, and outcomes

Study setting

9. Description of study settings (eg, community clinic, academic hospital) and list of countries where data will be collected. Reference to where list of study sites can be obtained

Settings: secondary schools

Country of data collection: Belgium, Hainaut province

Eligibility criteria

10. Inclusion and exclusion criteria for participants. If applicable, eligibility criteria for study centres and individuals who will perform the interventions (eg, surgeons, psychotherapists)

- School level

Inclusion criteria: secondary school, Hainaut province (Belgium)

- Adolescent level

Inclusion criteria: 3rd-4th grade in a recruited secondary school

Interventions

11a. Interventions for each group with sufficient detail to allow replication, including how and when they will be administered

- Control arm

The SILNE and SILNE-R studies have shown that, without specific intervention, school tobacco policies remain unchanged over time[9]. To give all participating schools the opportunity to improve their smoking situation, even schools assigned to the control arm will receive minimal intervention: feedback based on the evaluation conducted. This point was also important to motivate schools to participate in a study where they had only one chance in two to get an intervention. To achieve the smoke-free school environment goal, these schools are then left on their own.

- Intervention arm

Schools assigned to the intervention arm will benefit from the feedback and the advocacy coalition (school taskforce, Collaborative Learning and support Network, guidance) to achieve the smoke-free school environment goal.

11b. Criteria for discontinuing or modifying allocated interventions for a given trial participant (eg, drug dose change in response to harms, participant request, or improving/worsening disease)

Not applicable

11c. Strategies to improve adherence to intervention protocols, and any procedures for monitoring adherence (eg, drug tablet return, laboratory tests)

The audit: The audit will evaluate the school tobacco policy itself using a questionnaire completed by the principal. It will be addressed to the project coordinators of each school and covers the comprehensiveness, enforcement, and communication of the policy but also the provision of services, in the form of support to smoking cessation and smoking prevention activities.

11d. Relevant concomitant care and interventions that are permitted or prohibited during the trial

Not applicable

Outcomes

12. Primary, secondary, and other outcomes, including the specific measurement variable (eg, systolic blood pressure), analysis metric (eg, change from baseline, final value, time to event), method of aggregation (eg, median, proportion), and time point for each outcome. Explanation of the clinical relevance of chosen efficacy and harm outcomes is strongly recommended

- School level:

Implementation and integration of the STP. Questionnaires used in previous school-based tobacco prevention interventions will be used [25].

- Adolescent level:

Primary outcome: Social norm, assessed through the prism of descriptive and injunctive norm.

Secondary outcome: Smoking prevalence, assessed through the smoking status of the adolescents.

Participant timeline

13. Time schedule of enrolment, interventions (including any run-ins and washouts), assessments, and visits for participants. A schematic diagram is highly recommended (see Figure)

- Schools were invited to participate in the study by letter in May 2024.

- The evaluation will take place three times: at baseline (in September 2024), at one-year follow-up (in September 2025), and at two-year follow-up (in September 2026).

Sample size

14. Estimated number of participants needed to achieve study objectives and how it was determined, including clinical and statistical assumptions supporting any sample size calculations

School level: We will recruit a total of 20 schools: 10 for the experimental group, and 10 for the control group, similar to previous European studies on similar topics [15]

Adolescent level: Due to the clustering by school, the adolescent sample size must be inflated using a design effect so that school-specific smoking rates or school tobacco policy implementation can be considered. This design effect is equal to $1+(n-1)/\rho$ where n is the average school size (600 students in that province) and ρ is the intraclass correlation of smoking (0.05% in the SILNE study). As we will only evaluate adolescents from the 3rd and 4th academic years, we aim to survey 200 students per school. We expect a modest relative difference of 15% in smoking initiation between the experimental group and the control group, resulting in a total sample size of 2891 individuals.

<u>Recruitment</u>

15. Strategies for achieving adequate participant enrolment to reach target sample size

Schools were invited to participate in the study by letter in May 2024. The School Health Promotion (Promotion de la Santé à l'Ecole, PSE) and the education authority (Fédération Wallonie-Bruxelles) provided support in the dissemination of the invitation. After a few weeks, e-mails were sent as reminders to answer, then phone calls were made.

<u>Methods: Assignment of interventions (for controlled trials)</u>

Allocation:

<u>Sequence generation</u>

16a. Method of generating the allocation sequence (eg, computer-generated random numbers), and list of any factors for stratification. To reduce predictability of a random sequence, details of any planned restriction (eg, blocking) should be provided in a separate document that is unavailable to those who enrol participants or assign interventions

Randomisation follows a cross-stratification based on schools' location, to avoid contamination effects, and their social-economic index, as the social-economic status is strongly associated with smoking. *Figure 2* shows the flow chart displaying the research units per intervention arm.

<u>Allocation concealment mechanism</u>

16b. Mechanism of implementing the allocation sequence (eg, central telephone; sequentially numbered, opaque, sealed envelopes), describing any steps to conceal the sequence until interventions are assigned

Schools will be randomized using R studio. Randomization will be done after recruitment of the majority of the schools. Schools will be aware of their intervention after the first data collection. The content of the intervention (advocacy coalition) was only revealed to schools of the intervention group.

<u>Implementation</u>

16c. Who will generate the allocation sequence, who will enrol participants, and who will assign participants to interventions

Allocation sequence, schools enrolment and schools assignation was done by PL.

Blinding (masking)

17a. Who will be blinded after assignment to interventions (eg, trial participants, care providers, outcome assessors, data analysts), and how

Blinding is not possible in this intervention.

17b. If blinded, circumstances under which unblinding is permissible, and procedure for revealing a participant's allocated intervention during the trial

Not applicable.

Methods: Data collection, management, and analysis

Data collection methods

18a. Plans for assessment and collection of outcome, baseline, and other trial data, including any related processes to promote data quality (eg, duplicate measurements, training of assessors) and a description of study instruments (eg, questionnaires, laboratory tests) along with their reliability and validity, if known. Reference to where data collection forms can be found, if not in the protocol

The evaluation will take place three times: at baseline (in September 2024), at one-year follow-up (in September 2025), and at two-year follow-up (in September 2026). We will gather information in three different ways: an audit, a survey for the adolescents, and one for staff members.

- The audit will evaluate the school tobacco policy itself using a questionnaire completed by the principal. It will be addressed to the project coordinators of each school and covers the comprehensiveness, enforcement, and communication of the policy but also the provision of services, in the form of support to smoking cessation and smoking prevention activities.
- The adolescents' survey will be carried out following a repeated cross-sectional design. So, at each data collection, adolescents from the 3rd and 4th academic years will complete the survey. As adolescents will, during the 2 years of the intervention, move on through their academic course, survey participants will differ from year to year. This design is commonly used when an intervention aims to affect a community-level indicator of health.
- As teachers have been referred to as the main agents and change makers in school health programs, it is critical to get an insight into their perception of the school's duty to integrate health promotion into its policy. Also, social processes such as change agents, their relationships, and the context are important when implementing a program and will be assessed.

18b. Plans to promote participant retention and complete follow-up, including list of any outcome data to be collected for participants who discontinue or deviate from intervention protocols

Retention: To give all participating schools the opportunity to improve their smoking situation, even schools assigned to the control arm will receive minimal intervention: feedback based on the evaluation conducted. This point was also important to motivate schools to participate in a study where they had only one chance in two to get an intervention.

About deviation: Sensitivity analysis will also evaluate the association between the degree of implementation and the effect of the intervention

<u>Data management</u>

19. Plans for data entry, coding, security, and storage, including any related processes to promote data quality (eg, double data entry; range checks for data values). Reference to where details of data management procedures can be found, if not in the protocol

Data management plan was done on DMPonline (ID: 197261).

<u>Statistical methods</u>

20a. Statistical methods for analysing primary and secondary outcomes. Reference to where other details of the statistical analysis plan can be found, if not in the protocol

The effect of the advocacy coalition on primary and secondary outcomes will be analysed by multilevel regression models. Those account for the nested hierarchical structure of the data (per school). Subgroup analyses will be carried out to evaluate the differential effect of the intervention based on socio-demographic factors at the individual and at the school level. Sensitivity analysis will also evaluate the association between the degree of implementation and the effect of the intervention.

20b. Methods for any additional analyses (eg, subgroup and adjusted analyses)

Not specified yet.

20c. Definition of analysis population relating to protocol non-adherence (eg, as randomised analysis), and any statistical methods to handle missing data (eg, multiple imputation)

When implementing an intervention, not only the effectiveness of the intervention has to be assessed, but also the extent to which it has been implemented. Overlooking this could lead to a Type III error, known as a conclusion, that erroneously attributes observed findings to the intervention while this latter was not implemented as planned. Therefore, we will assess the fidelity of key elements of the intervention (the Task Force, the CLN, and the guidance)... Sensitivity analysis will also evaluate the association between the degree of implementation and the effect of the intervention.

<u>Methods: Monitoring</u>

<u>Data monitoring</u>

21a. Composition of data monitoring committee (DMC); summary of its role and reporting structure; statement of whether it is independent from the sponsor and competing interests; and

reference to where further details about its charter can be found, if not in the protocol. Alternatively, an explanation of why a DMC is not needed

There is not DMC because of the minimal risks related to the intervention.

21b. Description of any interim analyses and stopping guidelines, including who will have access to these interim results and make the final decision to terminate the trial

Such analyses are not planned because of the minimal risks related to the intervention.

<u>Harms</u>

22. Plans for collecting, assessing, reporting, and managing solicited and spontaneously reported adverse events and other unintended effects of trial interventions or trial conduct

No plan for this because of the minimal risks of the intervention.

<u>Auditing</u>

23. Frequency and procedures for auditing trial conduct, if any, and whether the process will be independent from investigators and the sponsor

Protocol changes will be discussed with the steering committee, in agreement with the funder.

<u>Ethics and dissemination</u>

<u>Research ethics approval</u>

24. Plans for seeking research ethics committee/institutional review board (REC/IRB) approval

Consent to participate in the study and to be randomised is given by the school principal, as is often the case in cluster randomised controlled trials. Regarding data collection, each person participating in a survey will give his/her consent by completing it.
The study got the ethical approval from the *Comité d'éthique hospitalo-facultaire UCLouvain* (Protocol n°: ADHAIRE).

<u>Protocol amendments</u>

25.Plans for communicating important protocol modifications (eg, changes to eligibility criteria, outcomes, analyses) to relevant parties (eg, investigators, REC/IRBs, trial participants, trial registries, journals, regulators)

No important modification will be possible without agreement from the funder. The study was accepted by the funder in its current form.

Consent or assent

26a. Who will obtain informed consent or assent from potential trial participants or authorised surrogates, and how (see Item 32)

The informed consent to participate in the surveys will be the starting point of the survey. Without it, the survey will not be available.

26b. Additional consent provisions for collection and use of participant data and biological specimens in ancillary studies, if applicable

Not applicable.

Confidentiality

27. How personal information about potential and enrolled participants will be collected, shared, and maintained in order to protect confidentiality before, during, and after the trial

No personal information will be required.

Declaration of interests

28. Financial and other competing interests for principal investigators for the overall trial and each study site

Authors have no competing interests as defined by Tobacco Prevention and Cessation, or other interests that might be perceived to influence the discussion reported in this article.

Access to data

29. Statement of who will have access to the final trial dataset, and disclosure of contractual agreements that limit such access for investigators

Only the investigators (VL, PL) will have access to the data.

Ancillary and post-trial care

30. Provisions, if any, for ancillary and post-trial care, and for compensation to those who suffer harm from trial participation

Not applicable.

Dissemination policy

31a. Plans for investigators and sponsor to communicate trial results to participants, healthcare professionals, the public, and other relevant groups (eg, via publication, reporting in results databases, or other data sharing arrangements), including any publication restrictions

Scientific publications based on baseline results and on longitudinal results are planned. Presentation to the steering committee and to authorities are also planned.

31b. Authorship eligibility guidelines and any intended use of professional writers

We will follow the authorship eligibility criteria as defined by the International Committee of Medical Journal Editors.

31c. Plans, if any, for granting public access to the full protocol, participant-level dataset, and statistical code

Not planned.

Appendices

Informed consent materials

32. Model consent form and other related documentation given to participants and authorised surrogates

Translated from the French:

"Before you start, please read the following carefully.

Your school is taking part in the ADHAirE project, which is being run by the Health and Society Research Institute (UCLouvain) in Hainaut. As part of the project, we'd like to ask you a few questions.

What is the questionnaire about?

This questionnaire is about you, your health and your school. It will be completed by around 4,000 pupils like you in Hainaut. The aim of the survey is to gain a better understanding of health behaviours, particularly smoking among teenagers. We want to hear about your experiences so that we can improve the health of young people today and in the future.

Are your answers anonymous?

Yes, we won't ask for your first or last name, and you don't have to write them down anywhere. This means that your data will remain strictly confidential, as laid down in the Law of 30 July 2018 on the protection of privacy. No one at your school, or in your family, will be able to see your answers. The research team will analyse your answers, but it will be impossible for them to know who answered which questionnaire.

Do you have to take part?

It is important that as many students as possible complete the questionnaire in order to collect good quality data. However, your participation is voluntary. You are free to answer or not to answer the questionnaire, as well as to end it at any time. No one will hold this against you."

Biological specimens

33. Plans for collection, laboratory evaluation, and storage of biological specimens for genetic or molecular analysis in the current trial and for future use in ancillary studies, if applicable

Not applicable.

© 2025 Laloux P. et al.
